# A Rare and Challenging Case of Neck Infection - Thyroid Abscess

**DOI:** 10.7759/cureus.15527

**Published:** 2021-06-08

**Authors:** Ashish Mishra, Muhammad Zohaib, Naved Muhammad Farooq, Syed Muhammad Hadi M Jah, Muhammad M Amjad, Ali Hussain

**Affiliations:** 1 Acute Medicine, Hull Royal Infirmary, Hull, GBR; 2 Internal Medicine, Hull Royal Infirmary, Hull, GBR; 3 Endocrinology, Hull Royal Infirmary, Hull, GBR

**Keywords:** thyroid abscess, ultrasound thyroid, bacteroides fragilis, acute suppurative thyroiditis, hashimotos thyroiditis

## Abstract

Acute suppurative thyroiditis (AST) is serious and rare infection of the thyroid gland, often it can progress to thyroid abscess. Both anatomical defects and underlying thyroid disorders are attributed to etiopathogenesis of the disease. Bacteria usually reach the gland either by lymphatic spread or via hematogenous routes. If untreated it has fatal outcome and had serious complications. The mainstay of treatment is usually a combination of intravenous antibiotics and drainage, and sometimes surgery.

## Introduction

Thyroid abscess is a rare pathology, with the incidence of less than 1% of all thyroid diseases. Their scarcity is attributed to the gland’s inherent protective factors, such as rich blood supply, high iodine content, capsular enhancement, and good lymphatic drainage [1]. Thyroid abscesses most often occur in patients who have pre-existing disorders of the thyroid gland, such as nodules or cancer; anatomic defects, such as pyriformis sinus fistula; or in patients with compromised immune system. Furthermore, distant infections may spread to the gland via haematogenous route as a result of bacteremia. We present a case of a patient who developed an abscess secondary to haematogenous spread from recent distant infection.

## Case presentation

A 57-year-old female presented to accident and emergency department (A&E) with five-day history of fever and new onset painful dysphagia to both solids and liquids. She denied any history of nausea, vomiting, sweating, weight loss and chills. She felt deep pain in the throat on swallowing associated with drooling of saliva and no hoarseness of voice or shortness of breath. A few weeks earlier she was admitted to hospital with perforated colon and treated with surgery and intravenous antibiotics. Her past medical history was significant for type 2 diabetes mellitus, hypertension, hyperlipidaemia and atrial fibrillation.

She had no history of neck trauma, pre-existent thyroid disease, or symptoms suggestive of upper respiratory tract infection. Her family history is significant for Hashimoto thyroiditis.

On arrival to A&E, her vital signs were stable (heart rate 93 beats per minutes (bpm), blood pressure 144/77 mm Hg, respiratory rate 18, saturation of oxygen (SpO_2_) 95% on air). Examination of the neck revealed left-sided thyroid swelling, 7 x 5 cm and ovoid in shape, hard on palpation and warm to touch. The swelling was immobile on swallowing or tongue protrusion, and there were no palpable cervical lymph nodes. Additionally, there was no retro sternal extension on both palpation and percussion. The rest of general and systemic examination were normal.

Her baseline blood investigations are shown in Table 1 which revealed anaemia (Hb 10.4) and raised C-reactive protein (CRP 131). Rest of investigation, urea, creatinine, electrolytes and liver function test were normal. Her thyroid function was abnormal and consistent with hyperthyroidism.

**Table 1:** Laboratory parameters for a patient on admission.

Lab parameter	Patient results	Normal range
Full blood count (FBC)		
-Hb	10.4	11.0-14.5 g/dL
-WBC	9.4	2.4-9.5 x 10^9^/L
-Platelets	334	150-450 x 10^9^/L
Inflammatory markers		
-CRP	131	<20 mm/hr
Liver Function Tests (LFTs)		
-Albumin	26	35-52 g/L
-Alkaline phosphatase	102	35-104 U/L
-Alanine Transaminase (ALT)	14	5-25 U/L
-Aspartate Transaminase (AST)	9	5-20 U/L
-Bilirubin	1.5	1-17 µmol/L
Thyroid function tests		
-Thyroid stimulating hormone (TSH)	0.03	0.27-4.20 mIU/L
-FT4	36.7	13.1-21.3 pmol/L
Renal Function Tests (RFTs)		
-Urea	1.5	2.8-8.1 mmol/L
-Creatinine	45	45-84 µmol/L
-Sodium	141	135-145 mmol/L
-Potassium	3.6	3.5-5.1 mmol/L
-Chloride	100	98-107 mmol/L

**Figure 1:**
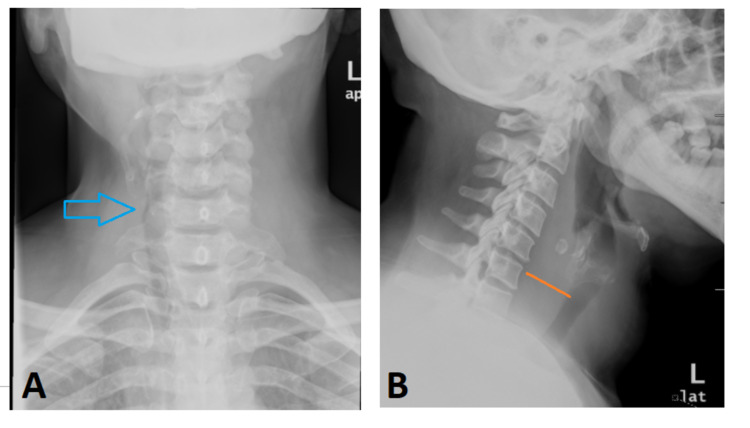
X-ray neck. (A) Anteroposterior view showed soft tissue mass seen mainly on the left side of the neck causing significant upper trachea deviation to the right side (arrow head). (B) Lateral view showed mass was extending from the anterior compartment to posterior compartment of the neck with abnormal widening of anterior paravertebral space (Orange line).

X-ray neck anteroposterior (AP) and lateral views (Figure 1A, 1B) were performed which showed soft tissue density/mass seen mainly on the left side of the neck causing significant upper trachea deviation to the right side. The mass was extending from the anterior compartment to posterior compartment of the neck with abnormal widening of anterior paravertebral space.

**Figure 2:**
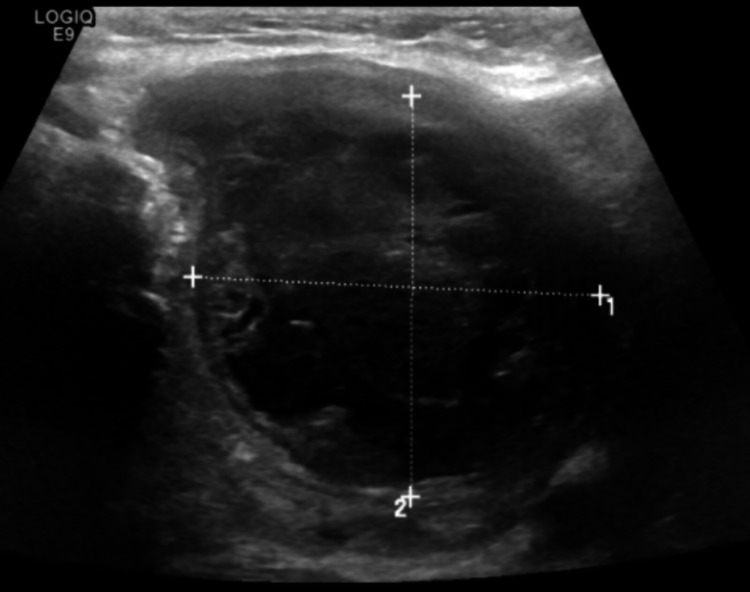
Ultrasound neck showed well-defined, large heterogeneous mass in left lobe.

Ultrasound neck (Figure 2) showed tender, well-defined, large 6.5 cm suspicious heterogeneous mass in the left lobe. The mass was causing significant upper trachea deviation to the right side. She was started on broad spectrum intravenous antibiotic Piperacillin with tazobactam (Tazocin) 4.5 gm three times per day (TDS) and analgesics for pain control.

**Figure 3:**
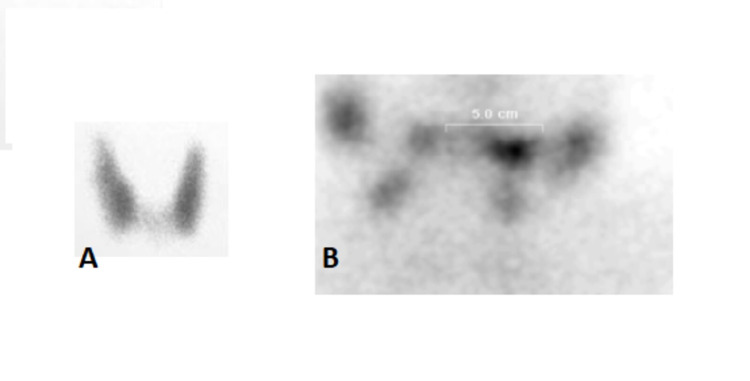
Thyroid scintigraphy. Image (A) shows normal scan and image (B) from the patient shows no thyroid uptake.

In a multi-disciplinary team (MDT) meeting, it was decided to do thyroid scintigraphy followed by computerized tomography (CT) of the neck. Thyroid scintigraphy (Figure 3A, 3B) did not show any uptake and overall gland was not visualized.

**Figure 4:**
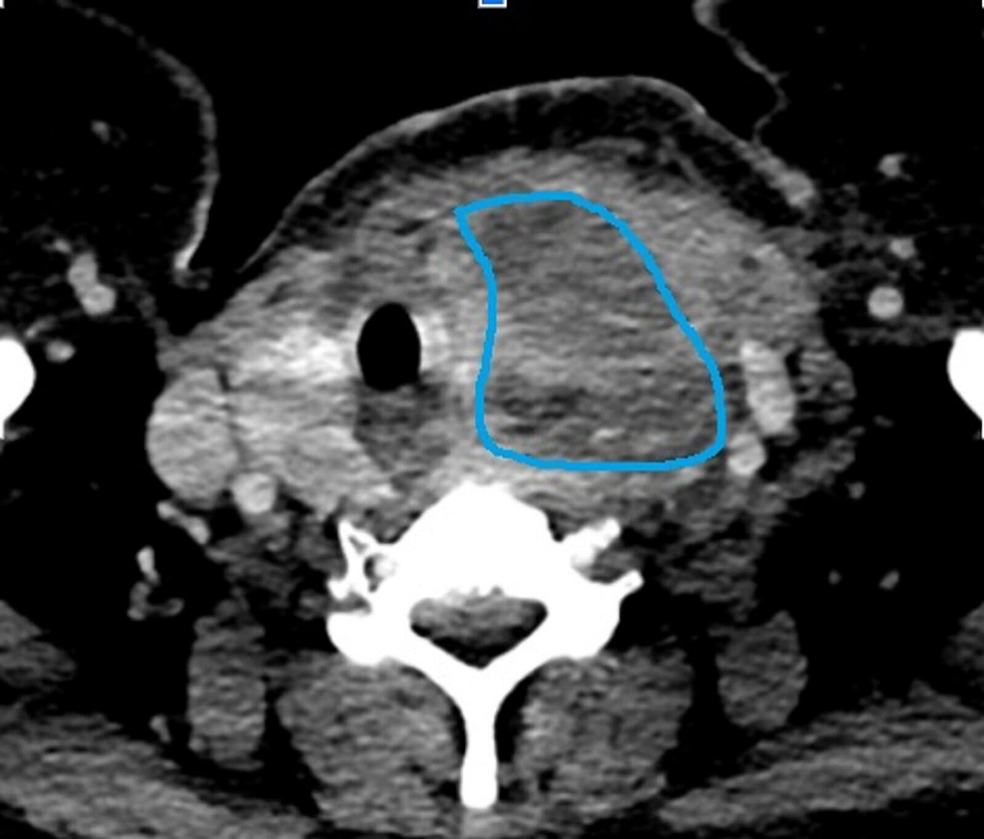
CT neck showed large thyroid mass with multiple hypodense area and fluid-filled content highly suggestive of necrosis or abscess (marked area). There was tracheal deviation.

Subsequently, CT neck (Figure 4) confirmed large thyroid mass with multiple hypodense area and fluid-filled content highly suggestive of necrosis or abscess.

On fine needle aspiration (FNA), 5cc of colloid was aspirated which on microbiology culture grew heavy growth of *Bacteroides fragilis*. Pain and dysphagia dramatically improved within 48 hours of intravenous (IV) antibiotics and her CRP also improved. She was treated conservatively with IV antibiotics for one week and no drainage or surgery was needed. She remained stable and discharged home on oral antibiotics. On subsequent follow-up after four weeks and three months she remained asymptomatic. In her case the probable source of bacterial seeding was her previous abdominal infection with* Bacteroides fragilis.*

## Discussion

Thyroiditis refers to a wide spectrum of inflammatory disorder. Acute suppurative thyroiditis is an uncommon form that is due to microbial infection. When the infection advances in patients with acute suppurative thyroiditis, an abscess may develop within the gland substance [2].

The diagnosis is usually delayed, since signs and symptoms are usually subtle with or without serious outcomes. Moreover, signs and symptoms of thyroid abscess usually mimic non-infectious inflammatory thyroid problems. It is essential to recognize the clinical and bacteriologic features of thyroid abscess for prompt treatment.

Most cases present with a sudden onset of pain and firm, tender, red, warm swelling in the anterior aspect of the neck that moves on swallowing as in our case [3]. Classically neck pain is unilateral and radiates to the ipsilateral mandible, ears, or occiput. Pain often worsens with neck hyperextension and improves with neck flexion. Other associated symptoms develop over days to weeks and may include fever with rigors and chills, sore throat, localized warmth and erythema. Likewise, compressive symptoms encompass hoarseness, dysphonia and dysphagia.

On examination, the thyroid gland is almost always swollen and tender. It can be unilateral or involves both lobes. Sometimes isthmus of gland is also involved. Rarely, a thyroid abscess can be manifested as a pulsatile mass [4]. Often localized lymphadenopathy and fluctuance of gland is seen.

Thyroid abscess should be differentiated from other more common thyroid conditions such as goitre, adenoma, intra-cystic haemorrhage. Furthermore, De Quervain's thyroiditis and rarely painful Hashimoto thyroiditis can present in similar fashion. Thyroid malignancy can masquerade as thyroid abscess and often poses diagnostic challenge.

The diagnosis of thyroid abscess can be supported by laboratory investigations. In general, majority of patients are biochemically euthyroid [5]. Leucocytosis, an elevated erythrocyte sedimentation rate (ESR), and elevated C-reactive protein (CRP) are usually present [6].

Lateral X-ray of neck may show evidence of tissue edema, and the tracheal air column may be deviated or compressed as in our case. Moreover, it can also help in categorizing the type of infection, for example presence of gas indicates anaerobic infections [7]. On the other hand, calcification may result from Echinococcus or Pneumocystis infection [8].

Ultrasound is very valuable for not only making diagnosis but can also give information about the spread to adjacent anatomical structures, which may be useful if surgical exploration is needed, and allows radiographically guided drainage of a thyroid abscess [9-10].

If ultrasound fails to establish the diagnosis or if the clinical course suggests extension of a thyroid abscess to other locations such as the mediastinum computed tomography (CT) and magnetic resonance imaging (MRI) scans of the neck may be warranted.

Abscess should be aspirated and sent for culture and staining to assist in the diagnosis and choice of proper antimicrobial therapy, as well as for cytology to ensure that a malignancy has not been missed [11]. The aspirate should be stained for bacteria, fungi, and mycobacteria and cultured for aerobic and anaerobic organisms. However, a negative Gram stain does not exclude bacterial infection [12].

Broad spectrum antimicrobial therapy is usually started empirically to cover a wide range of bacteria, at least until culture results are available.

Surgery may be needed to drain the infection and to repair any developmental abnormality that led to the development of abscess. Thyroid lobectomy may be needed in cases where extensive necrosis develops, or if the infection persists (as evidenced by leucocytosis, continued fever, and progressive signs of local inflammation) despite adequate antibiotics [10]. Alternative endoscopic approaches are acceptable, with similar success and a lower rate of complications [13].

Complications of thyroid abscesses are often fatal and carry grave prognosis. Complications include tracheal or oesophageal perforation, descending necrotizing mediastinitis, extension into the deep spaces of the neck, and death [14].

## Conclusions

Thyroid abscesses are rare and can present with common symptoms. Ultrasound is useful both in making diagnosis and to estimate the extent of abscess to localized structures. Furthermore, ultrasound-guided aspiration is needed for the culture and sensitivity of aspirate to tailor antibiotic treatment. In few cases, surgery and repair of the anatomical defect is needed. Owing to higher risk of complications and fatal outcome, clinicians should have high index of suspicion for thyroid abscess as a differential in patients who present with acute neck swelling.
